# A novel *SLC6A8* mutation associated with intellectual disabilities in a Chinese family exhibiting creatine transporter deficiency: case report

**DOI:** 10.1186/s12881-018-0707-5

**Published:** 2018-11-06

**Authors:** Qin Wang, Jingxin Yang, Yang Liu, Xingping Li, Fuwei Luo, Jiansheng Xie

**Affiliations:** Shenzhen Maternity and Child Healthcare Hospital, No. 3012, Fuqiang Road, Shenzhen, 518028 Guangdong China

**Keywords:** Creatine transporter deficiency (CRTR-D), X-linked, Intellectual disabilities, *SLC6A8*, Targeted exome sequencing

## Abstract

**Background:**

X-linked creatine transporter deficiency (OMIM#300036,CRTR-D) is characterized by cerebral creatine deficiency, intellectual disabilities, severe speech impairment, seizures and behavioral problems. Mutations in the creatine transporter gene *SLC6A8*, a member of the solute-carrier family 6 mapped to Xq28, have been reported to cause the creatine transporter deficiency.

**Case presentation:**

The proband presented at 5 yrs. 1 month of age with delays in intellectual and development, seizures and behavioral problems. A novel missense mutation, c.1181C > A (p.Thr394Lys), in the *SLC6A8* gene (NM_005629.3) was detected via targeted exome sequencing, and then validated by Sanger sequencing. Multiple in silico variant effect analysis methods, including SIFT, PolyPhen2, PROVEAN, and Mutation Taster predicted that this variant was likely damaging or diseasing-causing. This hemizygous variation was also identified in the affected brother with the same clinical condition and inherited from the heterozygous carrier mother. The diagnosis was suggested by increased urinary creatine/creatinine (Cr:Crn) ratio and markedly reduced creatine content peak by brain proton magnetic resonance spectroscopy (MRS). The proband’s mother became pregnant with a 3rd sibling, in whom the Sanger sequencing result of c.1181C > A was negative.

**Conclusion:**

The novel mutation c.1181C > A in the *SLC6A8* gene reported in a Chinese family has expanded the mutation spectrum of CRTR-D. The combination of powerful new technologies such as targeted exome sequencing with thorough systematic clinical evaluation of patients will improve the diagnostic yield, and assist in genetic counselling and prenatal diagnosis for suspected genetic disorders.

**Electronic supplementary material:**

The online version of this article (10.1186/s12881-018-0707-5) contains supplementary material, which is available to authorized users.

## Background

Creatine is essential in energy metabolism and plays a vital role in high-energy requirement organs such as muscle, brain and heart, as it facilitates the resynthesis of ATP from ADP through phosphocreatine [1, 2]. In humans, about half of the creatine is synthesized in the kidney and liver, and the other half is obtained by dietary intake. Although the brain is capable of producing minor amounts of creatine, the major proportion is taken up from the blood via solute carrier family 6 (neurotransmitter transporter, creatine), member 8 (*SLC6A8*) [2, 3]. Pathological mutations in *SLC6A8* affect the creatine transport into the brain and muscle. Creatine transporter deficiency (CRTR-D) (OMIM 300036) is the most common cerebral creatine deficiency syndrome (CCDS) and may be one of the leading causes of X-linked mental retardation (X-LMR) in males [4–6]. Clinical symptoms of creatine transporter deficiency in affected hemizygous males include intellectual disability (ID), development delay, expressive speech, and language delay, epilepsy, and autistic behavior [7–9]. Female carriers may exhibit mild cognitive impairment with behavior and learning problems [10]. The diagnosis of creatine transporter deficiency is based on clinical presentation and/or increased urine creatine/creatinine ratio, abnormal brain magnetic resonance spectroscopy and reduced brain creatine content. DNA analysis of disease-causing mutations in SLC6A8 or measurement of impaired creatine uptake in fibroblasts are confirmatory of the clinical diagnosis [11, 12].

The creatine transporter gene, *SLC6A8,* also known as CT1 or CRTR, is a member of the solute-carrier family 6 mapped to Xq28. The gene is expressed in most tissues and the creatine transporter (CT1) encoded by this gene is 635 amino acids long with 12 transmembrane domains and is required for the uptake of creatine in muscles and brain [6]. Defects in *SLC6A8* can result in X-linked creatine deficiency syndrome. The prevalence of *SLC6A8* deficiency is estimated at 0.8% to 2.1% [5, 13]. Since the first report of creatine transporter deficiency in 2001, many novel variants have been detected. The online database collecting the SLC6A8 variants has been established by Dr. GS Salomons (http://www.LOVD.nl/SLC6A8), which includes updated clinical and genetical data [14]. Currently, more than 140 SLC6A8 variants have been published.

Exome sequencing has become the most widely used targeting exome sequencing method, especially for monogenic (Mendelian) diseases [15]. Since exonic mutations cause the majority of monogenic diseases, the application of targeted exome panel sequencing has largely contributed to the identification of new disease-causing genes and confirmed its advantages in reducing the diagnostic odyssey for many suspect genetic disorders [16, 17]. In China, few CRTR-D patients have been reported. In this study, we present the detection of a novel mutation c.1181C > A (p.Thr394Lys) in the *SLC6A8* gene via targeted exome sequencing in a Chinese family with CRTR-D. The mutation was confirmed by Sanger sequencing. The detection of novel pathogenic variants in the exome by targeted exome sequencing has been a powerful and effective diagnostic tool in determining the molecular basis of genetic disorders.

## Case presentation

A male patient aged 5-year-and-1-month was referred for genetic evaluation of development and speech delay, intellectual disabilities at the genetic counselling clinic in Shenzhen Maternal and Child Healthcare Hospital. The parents described that an affected brother also presented the same clinical phenotype but was not available for the clinical examination. The chromosome karyotype and chromosomal microarray analysis (CMA) in the proband were normal. The mother was pregnant again and pursued genetic counseling. The proband was subject to comprehensive neurological testing including the Gesell Developmental index. Molecular genetic tests and biochemical and neurochemical analysis were performed on the proband. The present study was approved by the hospital’s Institutional Review Board and written informed consent was obtained from their parents.

### Clinical findings

**Fig. 1:**
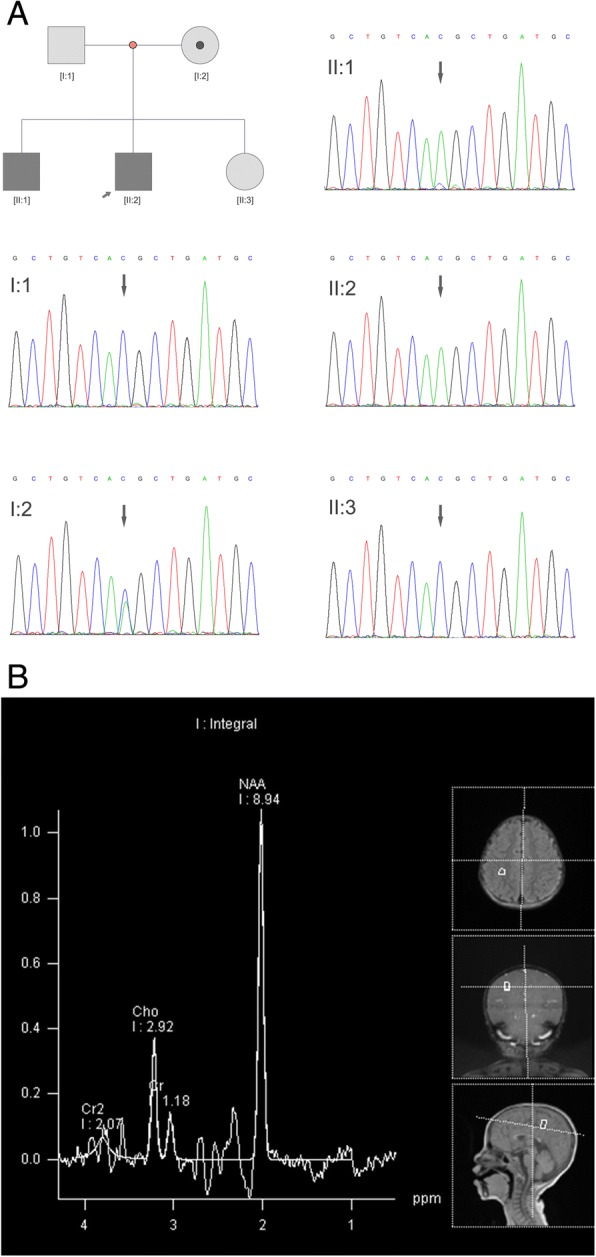
Molecular genetic tests and neurochemical analysis results in the index patient. **a**. Family tree of the proband and Sanger sequencing results in this study. Sanger sequencing validated the mutation, c.1181C > A (p.Thr394Lys), in the *SLC6A8* gene in family members as I:1 wildtype, I:2 heterozygous carrier, II:1 hemizygous, II:2 hemizygous and II:3 wildtype. **b**. Proton magnetic resonance spectroscopy (MRS), examination using a 3.0-T system on the brain showed marked reduction of the brain creatine peak (left part), and brain MRI showed a thin corpus callosum in the proband (right part)

The proband was the second boy of healthy nonconsanguineous parents (pedigree in Fig. 1a). He was born at 39 weeks of gestation from an uneventful pregnancy and delivered by Caesarean section (weight, 3600 g; length, 50 cm; head circumference, 36 cm). He showed head control at 12 months, ability to sit by himself at 15 months, and walking with aid at 20 months. His verbal language was nearly absent and he made no visual contact. He suffered from seizures from 6 months old. He had no craniofacial dysmorphism. Gastrointestinal problems such as chronic constipation or nausea were noted in the proband. The physical examination on the proband showed 95 cm height, weight 18.2 kg and developmental and language delay. The proband also had an electroencephalogram (EEG) test, which showed sharp and slow waves in sleep during 24-h EEG monitoring. A brain stem auditory-evoked potential (BAEP) test showed mild abnormality. The proband had a Children’s Autism Rating Scale (CARS) score of 33, which indicated mildly autistic characteristics. The Gesell developmental scale test was used to evaluate the proband. Both the development age (DA) and developmental quotient (DQ) data showed extremely low grades which suggested severe development delay (adaptability, DA = 14.23mo., DQ = 23; gross motor, DA = 26.37mo., DQ = 43; fine movement DA = 15.87mo., DQ = 26; vocabulary DA = 13.07mo., DQ = 21; personal-social skill DA = 13.3mo., DQ = 22). The test results are depicted in Additional file 1: Figure S1A. The affected brother of the proband (II:1) was not available for the physical examination. The parental description of the clinical phenotype of the brother was mostly the same as the proband. The parents were physically healthy and indicated no significant past medical, surgical or family history.

### Methods and results

DNA samples were provided from the index patient and other family members, which were extracted as previously described [18]. The present study used the TruSight One Sequencing Panel and NextSeq 500/550 Mid Output v2 kit (300 cycles) with high depth of coverage for 4813 target genes (approximately 62000 target exons) that are associated with clinically relevant phenotypes. An average sequencing depth of 136.88x was achieved and 98.25% of targeted variants were covered at least to a 10x depth, and 97.04% of targeted variants were covered at least by 20x. The total detected variants numbered 24594, which included 21,733 SNPs, 1,182 insertions and 1,679 deletions respectively. The data were analyzed on the TGex (Translational Genomics Expert) platform featuring with the VarElect scoring system [19]. A missense mutation, c.1181C > A (p.Thr394Lys), in the *SLC6A8* gene was called with high probability as a candidate mutation.

Sanger sequencing was performed to confirm the *SLC6A8* gene c.1181C > A mutation (forward primer 5’ ACGGAACTTGTCAGATTGT3’, and reverse primer 5’CAACAGCATGAAGAAGAACA3’). The father (I:1) was wildtype and the mother (I:2) was heterozygous for the c.1181C > A variation. The affected brother (II:1) and the proband (II:2) both carried the hemizygous variation of c.1181C > A. The pregnant mother had an amniocentesis at 22 weeks and Sanger sequencing targeting the *SLC6A8* gene c.1181C > A was performed. The result showed a wild-type allele (II:3) and the mother gave birth to a healthy baby girl (Fig. 1a). In silico variant prediction analysis methods, including SIFT, PolyPhen2, PROVEAN, and Mutation Taster demonstrated this variant had probably damaging or diseasing-causing effects.

### Biochemical and neurochemical analysis

Biochemical screening was performed with blood and urine samples from the proband and his mother. The creatine/creatinine (Cr:Crn) ratio was determined by liquid chromatography-mass spectrometry with deuterated internal standards in two urine samples taken on different days. A urine creatine test of the proband showed significantly elevated levels of creatine (0.805 mg/ml, normal control 0.160 ± 0.177 mg/ml) (Additional file 1: Figure S1B), and the creatine/creatinine ratio was significantly elevated compared to controls. Proton magnetic resonance spectroscopy (MRS, Magnetom Skyra 3.0-T, Siemens Healthcare GmbH, Erlangen, Germany), examination using a 3.0-T system at the brain left parietal lobe, right parietal lobe and genu of corpus callosum all showed marked reduction of the brain creatine peak (Fig. 1b left part). Brain MRI showed a thin corpus callosum in the proband (Fig. 1b right part). The MRS and MRI examination of the mother (I:2) showed normal results (Additional file 1: Figure S1C).

## Discussion

In the present study, targeted exome sequencing revealed a novel missense mutation, c.1181C > A (p.Thr394Lys), in the *SLC6A8* gene (NM_005629.3), resulting in CRTR deficiency syndrome in a Chinese family. The index patient was referred for unexplained intellectual disability, with severe speech delay, seizures and autistic behaviors. High-throughput sequencing was performed on the proband, and the results revealed a pathogenic missense mutation in the creatine transport gene *SLC6A8*. The missense variation c.1181C > A was in the predicted transmembrane domain 8 and in silico analysis predicted this variant as possibly damaging. The diagnosis of CRTR-D was confirmed by the urinary creatine/creatinine measurement and MRS of the brain. The hemizygous index male patient presented with the characteristic phenotype comprising intellectual disability, severe speech delay, epilepsy, and behavioral disturbances. The hemizygous brother was identified through candidate variant analysis, and his clinical features were identical to the proband, according to the parental reports. Gastrointestinal problems such as vomiting and constipation were also noted in both brothers. Neither brother had obvious craniofacial dysmorphism. The clinical presentations are similar to the previous clinical reports of CRTR-D patients [20]. The proband’s heterogenous mother was physically normal. The proband’s mother became pregnant with a 3rd sibling, on whom amniocentesis and Sanger sequencing result of c.1181C > A was negative.

CRTR-D is reported to be the most frequent disorder of creatine synthesis and cellular transport and is supposed to be one of the more common causes of X-linked mental retardation (XLMR) [5, 20]. The diagnosis of CRTR-D includes the clinical presentation of mental retardation (MR), expressive speech and language delay, epilepsy, developmental delay and autistic behavior in affected males. Laboratory hallmarks include an increased urinary creatine/creatinine (Cr:Crn) ratio, which is a widely available test. A reduction of the creatine signal in the proton magnetic resonance spectroscopy (H-MRS) of the brain is also a very sensitive screening method, although H-MRS is not regularly equipped in many centers. Additional molecular genetic testing in the form of the sequencing of the *SLC6A8* gene is necessary to confirm the diagnosis. Next generation sequencing will play more roles in identifying the genetic causes of CRTR-D. The creatine uptake test in cultured fibroblasts should be studied to prove the pathogenicity in case of a novel unclassified variant. The estimated frequency of *SLC6A8* deficiency in males with XLMR is 2.1%,1.5% and 0.8% in different studies. The prevalence in family cases is 5.4% [5, 13, 21]. The estimated carrier frequency of CRTR-D is 0.024% in females in the general population [22]. A total of 142 *SLC6A8* variants have been reported in 667 individuals (according to the LOVD SLC6A8 database), which mainly in Western populations. All known mutations are either de novo (30%), or maternally inherited (70%) [4]. Phenotypic variability has been observed in male patients, from mild to severe clinical symptoms, which may depend on the location of the missense mutation in the protein structure [7, 8, 23, 24]. In our study, the two siblings had the same hemizygous mutation, which was maternally inherited. They were severely affected, and clinical follow-up should be done as previous reports document progressive cerebral atrophy and the broader clinical presentation of *SLC6A8* deficiency in reported adult patients [25, 26]. Since the diagnostic guidelines of CRTR-D have been well established, more patients should be recruited to study the genotype-phenotype correlation and mutation spectrum in Chinese for its high frequency in patients with unexplained mental retardation.

Currently CRTR-D is an untreatable disease and the pathogenic mechanism is not entirely clear. Recent evidence proposed the new role of creatine as neuromodulator or neurotransmitter based on the prediction that cerebral creatine deficiency in CRTR-D derives from a failure of creatine recycling following release [27]. Creatine depletion in the brain may reduce the white matter brain volume and thus affect the cognitive ability [28]. A mouse model of CRTR-D has revealed that Cr deficiency in the cerebral cortex and hippocampus regions is apparent in the cognitive defects. Endogenous uptake of creatine does not compensate for the loss of creatine in the mouse or human brain [29, 30]. RNA sequencing has suggested that *SLC6A8* mutation will result in gene expression alterations, especially genes encoding components of the extracellular matrix or cell structure. Dysfunctional extracellular matrix molecules in *SLC6A8*-deficient neurons are related to neurodegenerative disorders, learning and epilepsy [31]. The restoration of the extracellular matrix could become a therapeutic breakthrough for CRTR-D.

## Conclusion

In conclusion, a novel missense mutation, c.1181C > A (p.Thr394Lys), in the *SLC6A8* gene was detected in a Chinese family through targeted exome sequencing. The diagnosis of CRTR-D was confirmed by urinary creatine/creatinine measurement as well as MRS of the brain. The combination of targeted exome sequencing with systematic clinical evaluation of patients used in suspected genetic disorders may improve diagnostic yield, assist in the medical care of patients and offer genetic counseling and prenatal diagnosis for family members.

## Additional file

Additional file 1: **Figure S1.** A. Gesell developmental scale evaluated the proband as severely developmentally delayed. B. Urine creatine was significantly increased (0.805 mg/ml) in the proband (peak height 25,000, upper left) compared with the normal control (NC) value of 0.160 ± 0.177 mg/ml (peak height 3,200, below left). The internal standard peak is the reference for the test at right. C. Proton magnetic resonance spectroscopy (MRS), examination using a 3.0-T system on the brain showed normal brain creatine peak (left part) and brain MRI showed normal corpus callosum in the proband’s mother (right part). (TIF 5633 kb)

## Abbreviations

CCDS: Cerebral creatine deficiency syndrome
Cr:Crn: Creatine/creatinine
CRTR-D: Creatine transporter deficiency
DA: Development age
DQ: Developmental quotients
H-MRS: Proton magnetic resonance spectroscopy
ID: Intellectual disability
MRI: Magnetic resonance imaging
SLC6A8: Solute Carrier Family 6 (Neurotransmitter Transporter, Creatine), Member 8
X-LMR: X-Linked mental retardation
